# Localization of spleen and kidney organs from CT scans based on classification of slices in rotational views

**DOI:** 10.1038/s41598-023-32741-y

**Published:** 2023-04-07

**Authors:** Tomasz Les, Tomasz Markiewicz, Miroslaw Dziekiewicz, Jaime Gallego, Zaneta Swiderska-Chadaj, Malgorzata Lorent

**Affiliations:** 1 University of Technology, Plac Politechniki 1, 00-661 Warsaw, Poland; 2 Military Institute of Medicine, Szaserów 128, 04-141 Warsaw, Poland; 3 University of Barcelona, Gran Via de les Corts Catalanes, 08007 Barcelona, Spain

**Keywords:** Kidney diseases, Computer science

## Abstract

This article presents a novel multiple organ localization and tracking technique applied to spleen and kidney regions in computed tomography images. The proposed solution is based on a unique approach to classify regions in different spatial projections (e.g., side projection) using convolutional neural networks. Our procedure merges classification results from different projection resulting in a 3D segmentation. The proposed system is able to recognize the contour of the organ with an accuracy of 88–89% depending on the body organ. Research has shown that the use of a single method can be useful for the detection of different organs: kidney and spleen. Our solution can compete with U-Net based solutions in terms of hardware requirements, as it has significantly lower demands. Additionally, it gives better results in small data sets. Another advantage of our solution is a significantly lower training time on an equally sized data set and more capabilities to parallelize calculations. The proposed system enables visualization, localization and tracking of organs and is therefore a valuable tool in medical diagnostic problems.

## Introduction

Abdominal diseases are a heterogeneous and extensive group of diseases. The most common include renal diseases such as chronic kidney disease, glomerulopathy, nephritis, kidney stones, renal cysts, and tumors^1^. Many kidney diseases may be asymptomatic for many years, or symptoms may be vague. Similarly, symptoms of a diseased organ can be easily missed because some abdominal organs have no sensory receptors and do not hurt. Similarly, diseases of the spleen such as splenomegaly are caused by the presence of the following diseases: Infectious, metabolic, storage and cancerous diseases. Imaging studies of the abdomen are one of the most important tools in the medical diagnosis of diseases of the abdomen. One of the most accurate examinations is computed tomography (CT), which uses X-rays to provide a detailed view of a section through the abdominal cavity^2,3^. Computed tomography of the abdomen can take hundreds of X-ray images from different angles and sides. The images are then overlaid with appropriate software to create accurate models of the scanned organ. Then, a specialist, such as a radiologist, manually does through all the images to find lesions. Since the number of slices for a patient can be several hundred, a thorough review of all images is a time-consuming task. In addition, human error can be caused by a variety of factors such as lighting, technical conditions, or fatigue. In contrast, computer systems that perform image analysis are not immune to such problems. They perform this task much faster than humans and are now an indispensable tool for the work of specialists.

### Problem statement

**Figure 1:**
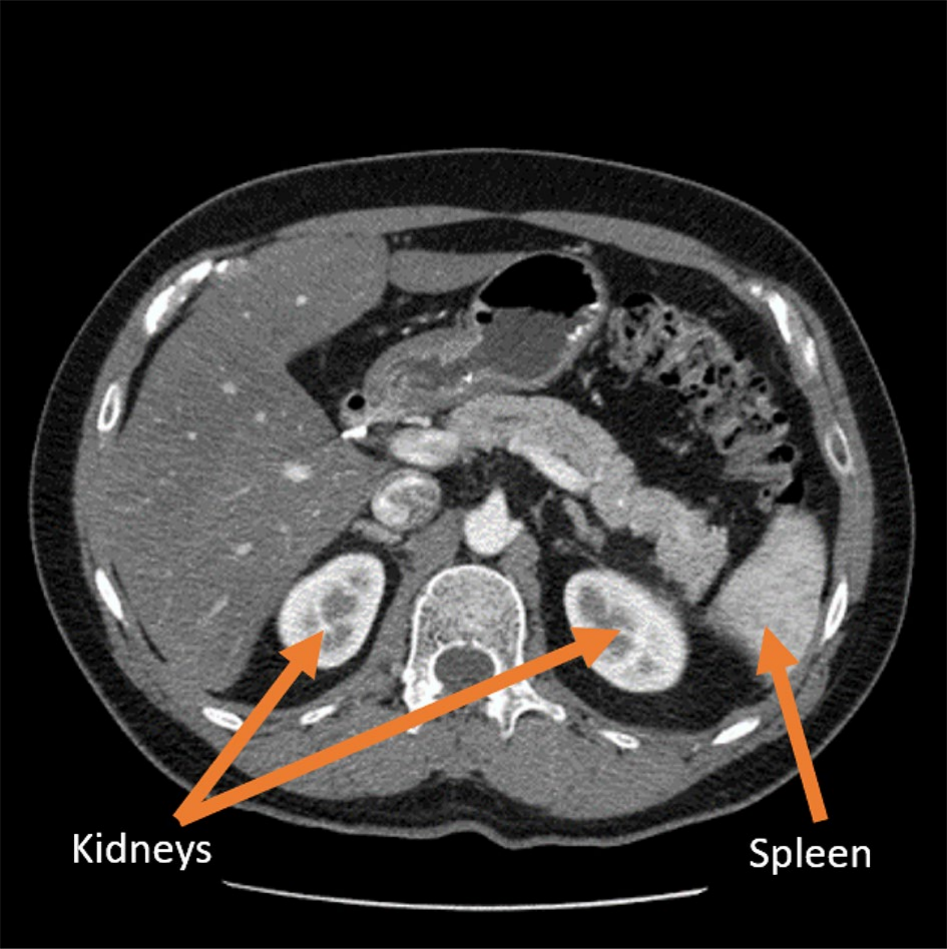
Example of a selected original CT section. The orange arrows indicate the labeled organs marked by an expert: Kidneys and Spleen, which are in the area of interest of the study.

Diagnosis of kidney disease is a multistep process. Basic diagnostics include laboratory tests and imaging studies, including ultrasound (USG), abdominal radiographs, urography, angiography, computed tomography (CT), magnetic resonance imaging (MRI), and radioisotope scans. Each type of examination provides specific information and allows better differentiation of selected areas. For the evaluation of renal tumors, the gold standard is computed tomography, which can determine the enhancement in renal masses by comparing Hounsfield units (HU) before and after contrast administration^4^. Abdominal examination CT also provides information to assess the course of many diseases, such as determining lymph node status, involvement of the adrenal glands and other solid organs, renal function and morphology, and extent of primary tumor and venous involvement^5^. Computed tomography is a method of producing tomographic images (cross-sections) of the object under study. It uses a combination of projections of the object from different directions to produce cross-sectional (2D) and spatial (3D) images. It provides images with high resolution. If hematuria is suspected and calcifications are being investigated, non-contrast tomography can be performed. To visualize the size and outline of the kidneys as well as parenchymal changes such as tumors and cysts, an examination with contrast medium is required^6^. The image in an X-ray examination CT is reconstructed in a pixel matrix, which is usually 512 $\times$ 512 pixels. In a typical CT examination, about 60–100 cross sections contain the examined organ. The number of sections obtained depends on the scanner parameters and resolution. An example of a selected CT section for a particular patient is shown in Fig. 1.

**Figure 2:**
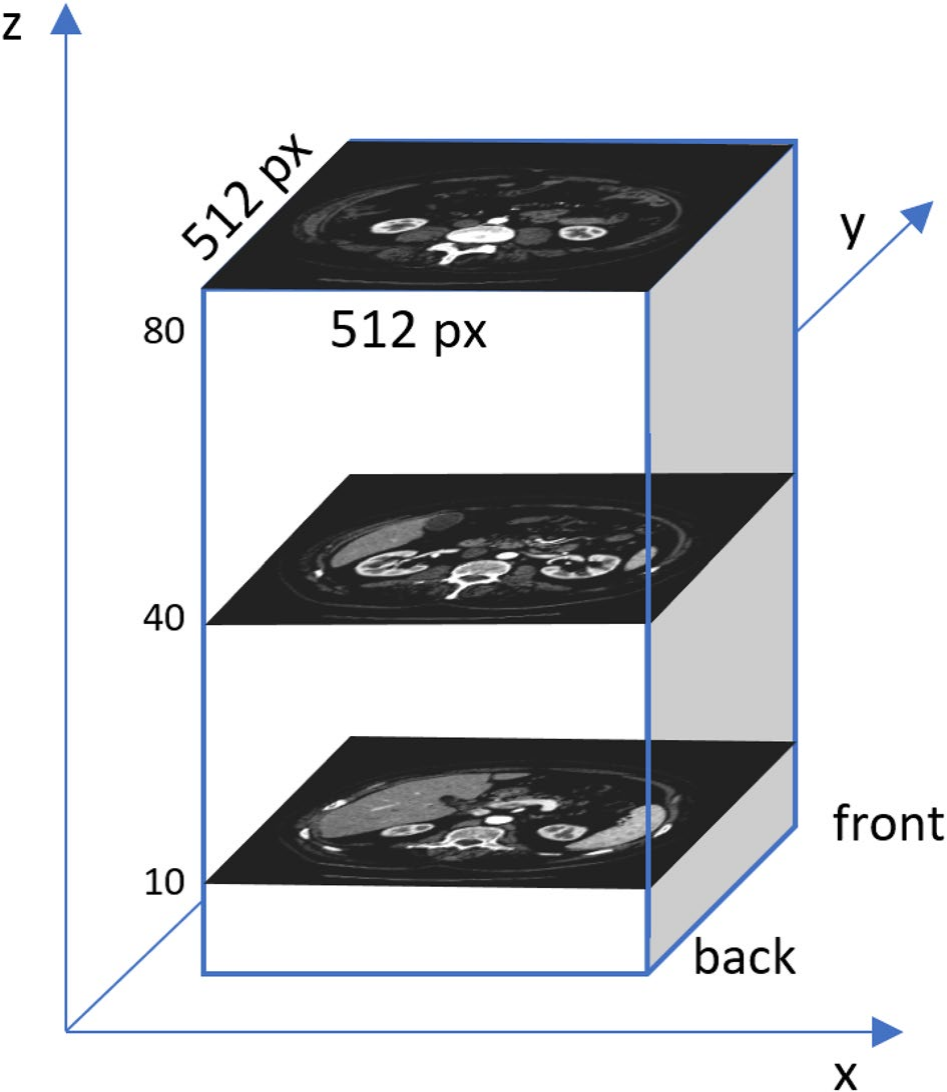
Diagram showing the position of the selected slices with indices 10, 40 and 80 in the volume, together with superimposed X–Y–Z axes.

In this figure, the image has been corrected by automatically adjusting the image brightness to improve the visibility of each organ. Figure 2 shows a schematic representation of the position of slices (**a**), (**b**) and (**c**) with the superimposed X–Y–Z axes in the abdominal segment.

Figure 2 shows (a) the lower section at index: 10, (b) the middle section: at index 40, and (c) the upper section: at index 80. The visible areas differ in size and shape. Radiographic evaluation is performed by specialists to locate and identify the nature of the lesion. Analysis of a single case often requires the operator to inspect each slice manually. It should be emphasized that the number of slices can be significantly higher when the examination includes more than just the abdomen, for example, in whole-body scans, which are often used to assess injuries^7^. Currently, numerous studies are focusing on the development of computer-assisted techniques that facilitate the localization of organs in multiple slices and speed up the diagnosis. Special attention is given to systems that can automatically contour the organ. In the diagnosis of kidneys and spleen, there are two main tasks: localization and classification. Localization of the organ is an essential step that allows the radiologist to quickly find suspicious areas and is also required prior to classification to determine which organ or cancer lesion we are dealing with. In fully automated systems that do not require human intervention, the quality of organ detection later determines the quality of classification. Computational image processing techniques for organ localization use segmentation techniques based on morphological image processing and machine learning^8–10^. Classification techniques are a separate task based mainly on machine learning—currently mainly on Deep Learning^11,12^. In this paper, we propose an innovative method for kidney and spleen region detection in CT images. Unlike other proposed solutions for kidney region recognition, we propose a solution for organ contour recognition without semantic segmentation based on Deep Learning network for region classification and clustering. The proposed technique automatically detects organs by analyzing the entire abdominal segment without the need for initial manual segmentation.

In recent years, a considerable number of solutions and prototype systems for CT organ segmentation have been developed. The main trend is semantic segmentation based on deep neural networks and pre-and post-processing^11,13–18^. Reported diagnostic accuracy values are in the range of 85–97% for organ localization. Commonly used accuracy measures are F-score, F-measure or F1-score, also known as Sørensen–Dice coefficient or Dice similarity coefficient (DSC). Semantic segmentation uses special networks adapted for this purpose. A predominant option in this category is U-Net^19^, which the authors used to achieve 97% accuracy in the KiTS 2019 Challenge^20^. U-Net and other modifications like Unet++^15^, Dense U-Net^21^, Inception U-Net^22^ is a network specifically designed for the task of segmenting medical images. However, other proposed networks such as ResNet^23^ or V-Net^24^ also provide satisfactory segmentation results. Research on new solutions for kidney segmentation techniques is currently limited to testing new network parameters, architectural changes and the introduction of new pre-and post-processing solutions. Some works present a system based on a CNN network where the detection modules are cascaded^25–27^. A good example is the proposal for a cascaded system consisting of a recognition module using the VGG-16 model and a segmentation module^28^. In general, localization is a simpler problem than segmentation. The successes of region methods, especially Region Proposal Networks (RPNs), have shown the effectiveness of the mechanism in object detection^27^. Very good results in data segmentation are limited by high system requirements (especially the learning process), high computational complexity, and the need to prepare a potentially large set of learning data. Not every neural network can be easily used for semantic segmentation. In this paper, we present a new approach to the problem of segmenting kidneys and spleens using classification networks. This completely different approach allows the use of a new set of classifiers and does not require such a large set of learning data.

## Methods

All methods were performed in accordance with the relevant guidelines and regulations. Methods were performed on archived 90 CT images, made with a contrast medium of patients with kidney or spleen disease. The images were collected in association with the Military Medical Institute (Warsaw, Poland). Each scan was annotated with spleen/kidney contours by a surgeon with oncological experience. Final diagnoses were confirmed by histopathological examination. All data used in the study has been anonymised and there is no possibility of associating the study with a specific human. No additional human studies were required to complete the presented experiments.

**Figure 3:**
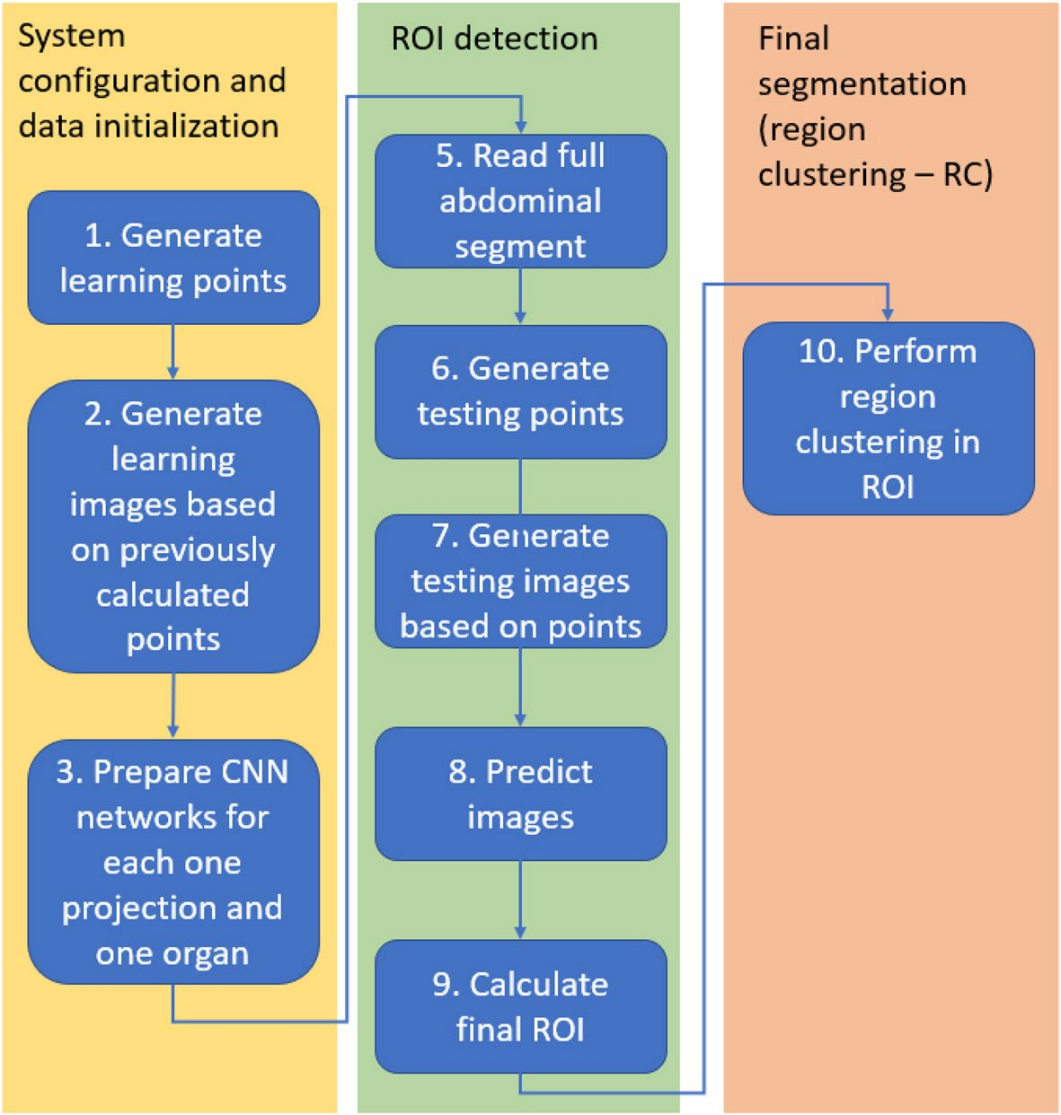
Workflow of the proposed system. The system consists of three main steps, from CT scan of the body to ROI and RC recognition: (1) system configuration and data initialization, (2) ROI detection (3) final organ segmentation.

The proposed solution is divided into two steps: the detection of the region of interest (ROI) and the classification of the region (RC). The explanation of the solution is given for the right kidney (CT images are most often presented as a cross-section and, due to the positioning of the patient on the X-ray table, the right kidney is visible on the left side in radiological imaging). For the other kidney and the spleen, the algorithm is the same. The kidney and spleen are three-dimensional structures, and the first step is to find a cube with the smallest volume that contains the whole organ (ROI). The second step is to cluster the regions within the found ROI per cube into two classes: kidney/spleen and background. The workflow of the proposed system to find the ROI and the RC from the scans of the body CT is shown in Fig. 3.

As shown in Fig. 3, the first step of the proposed system involves converting the CT body scan into a region of interest, followed by organ recognition based on the predefined ROI. The ROI recognition step requires automatic decomposition of the body scans into slices in three spatial dimensions X, Y and Z.

Then, classification of particular slices is performed using three CNN networks. Separate CNN’s models were prepared for a different organs (renal or spleen). The proposed method is based on classifying individual slices in different spatial dimensions X,Y and Z. By performing the classification through three networks for each dimension, it is possible to find three surfaces strictly delimiting the area of renal or spleen respectively.

The combination of the results allows the definition of the ROI. Region clustering is then performed within the ROIs to find the areas related to the kidney or spleen. According to Fig. 3, steps 1 and 2 are presented in “Recognition of the region of interest” and “Generation of learning data”, respectively. Step 3 is presented in “Learning process based on CNN network”. Steps 5–9 are presented in “Generation of test data”.

### Recognition of the region of interest

**Figure 4:**
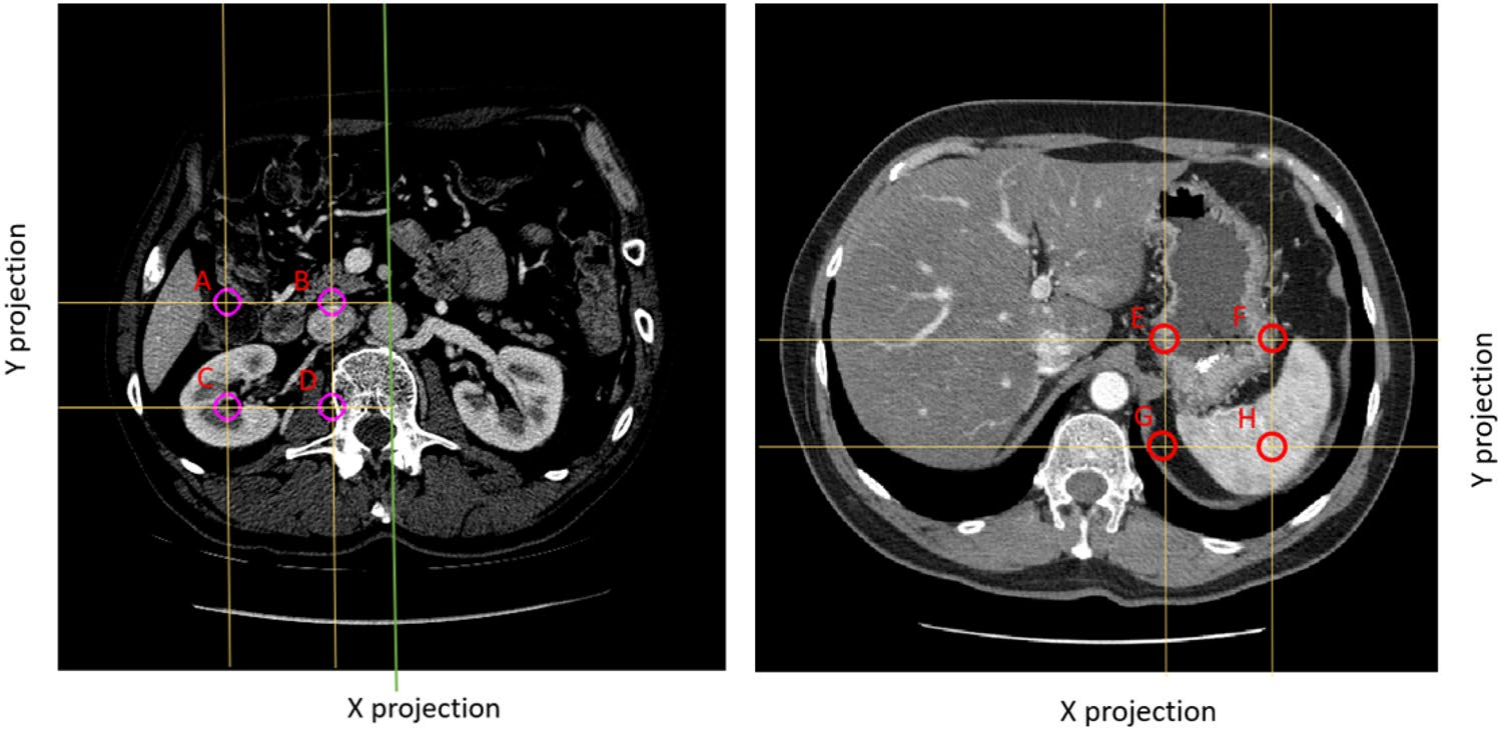
Example CT scan with superimposed points A, B, C, and D and intersecting axes in the X and Y axes. The green line crosses the center of the image.

Detecting of the region of interest as a 3D cube containing the entire kidney/spleen is a crucial task because the accuracy of the ROI calculation determines the future course of kidney detection. Many reference methods for finding ROI are based on morphological operations, noise reduction with Gaussian low-pass filter, texture analysis by computing the local entropy of the image, threshold selection and object windowing^29,30^. We propose a solution using a machine learning technique based on three convolutional neural networks (CNNs). Each network performs recognition in a different X, Y, and Z projection. An example of a slice in Z projection is given in Fig. 4. Each point *P*(*x*, *y*, *z*) in the three-dimensional space can be classified as a point belonging to the ROI or the background. Figure 4a shows four points (*x*, *y*) :  *A*(210, 230), *B*(130, 230), *C*(130, 310), *D*(210, 310), where $z=20$ for all points. The points E–H in Fig. 4 for the spleen were determined using a similar approach.

**Figure 5:**
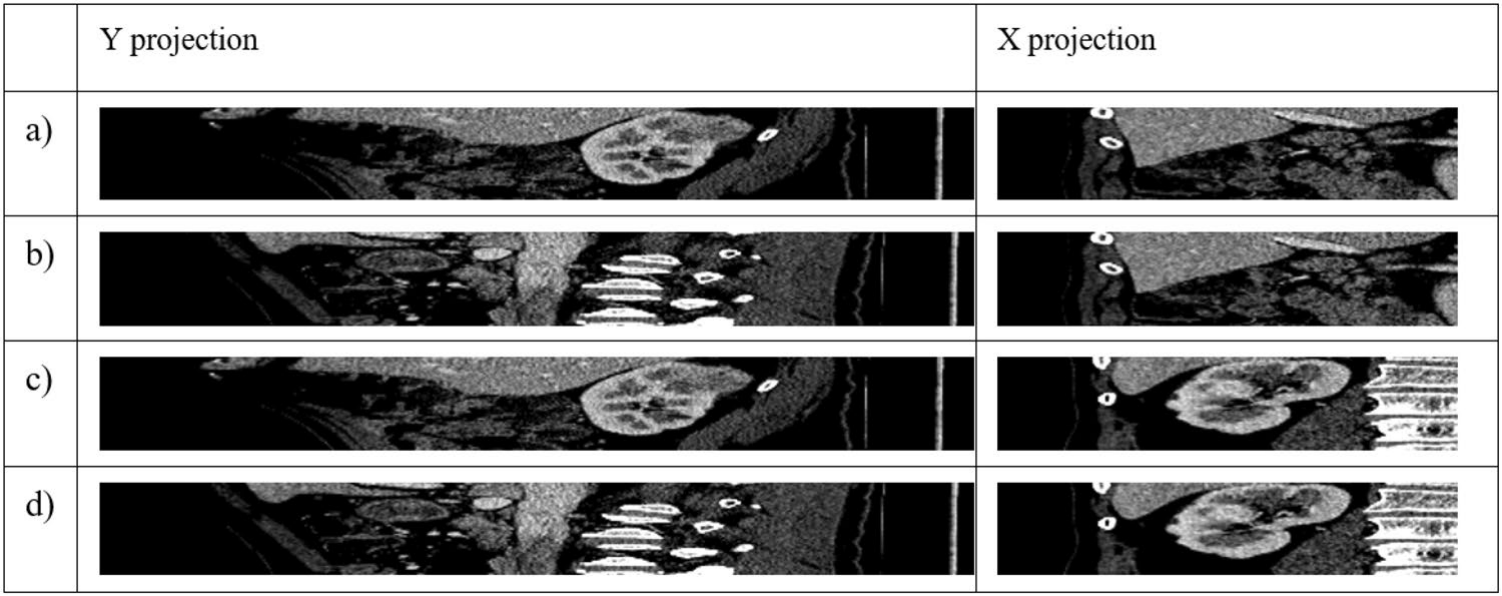
Cross-sections of the X and Y axes for points A, B, C and D, as shown in Fig. 4.

In Fig. 4a, intersection lines are drawn on the X and Y axes for each point. Point A does not intersect the kidney on the Y axis; it intersects the kidney on the X axis. Point B does not intersect the kidney on the X and Y axes. Point C intersects the kidney on both the X and Y axis. Point D intersects the kidney on the Y axis but not on the X axis. The X axis was shortened to half so that the other kidney was not visible. Four cuts were then made along the X axis and Y axis (yellow lines) at points A, B, C, and D in Fig. 4a. Figure 5 shows the slice in X and Y projection.

As we can observe in Fig. 5, only point C shows a kidney in both the X and Y projections. Any point for which three views in the X, Y and Z axes are classified as a kidney image belongs to the ROI. The classification of the images was performed using a convolutional network, as described in “Learning process based on CNN network”.

### Process of dataset creation

According to the assumption made in “Recognition of the region of interest”, all points of the image should be checked to determine the ROI area . For a typical 512 $\times$ 512 pixel CT scan, there are 262,144 points. This is clearly too many points, and point generation should be optimized for both the learning and test datasets. “Generation of learning data” presents the algorithm for generating the learning data. Section “Learning process based on CNN network” presents the CNN network used and its parameters. Section “Generation of test data” presents an algorithm for generating points for the test data.

### Generation of learning data

**Figure 6:**
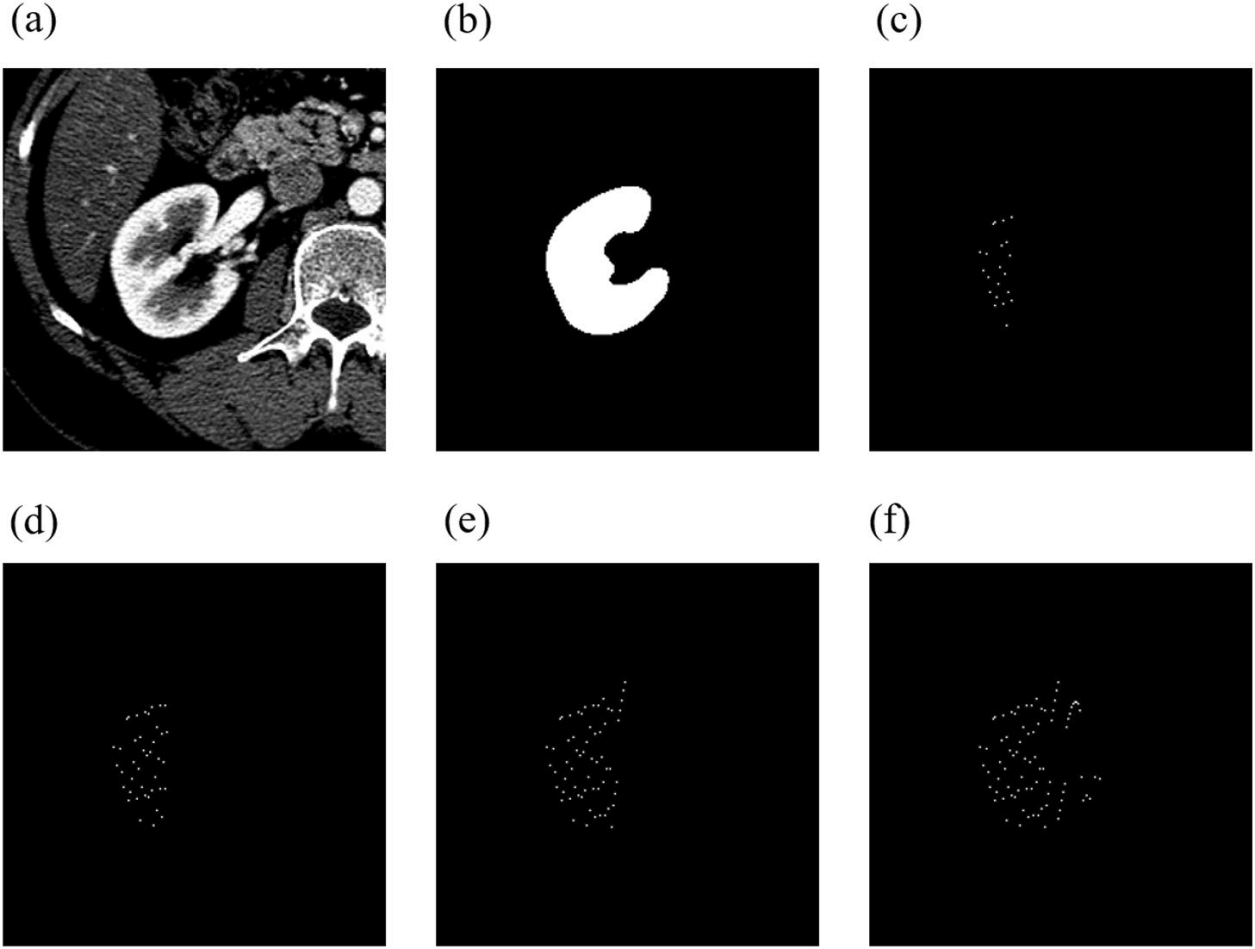
The process of generating points for the class OC. (**a**) shows a fragment of the original CT image. (**b**) shows a mask with a kidney labelled by an expert. (**c**–**f**) shows the point generation process.

The proposed solution is based on two classes: the organ class (OC) (for kidney/spleen) and the background class (BC). Figure 6 shows the process of generating learning points in kidney recognition for the class OC.

Figure 6 shows that the entire process of generating points for the kidney class only generates learning points within the range of the expert mask. The selected points are equidistant (every 50 px) from each other along the Y axis. This type of point generation allows for a significant reduction in learning data while maintaining regular point selection. To generate the points for the BC class, an algorithm was developed whose main steps are:

**Figure 7:**
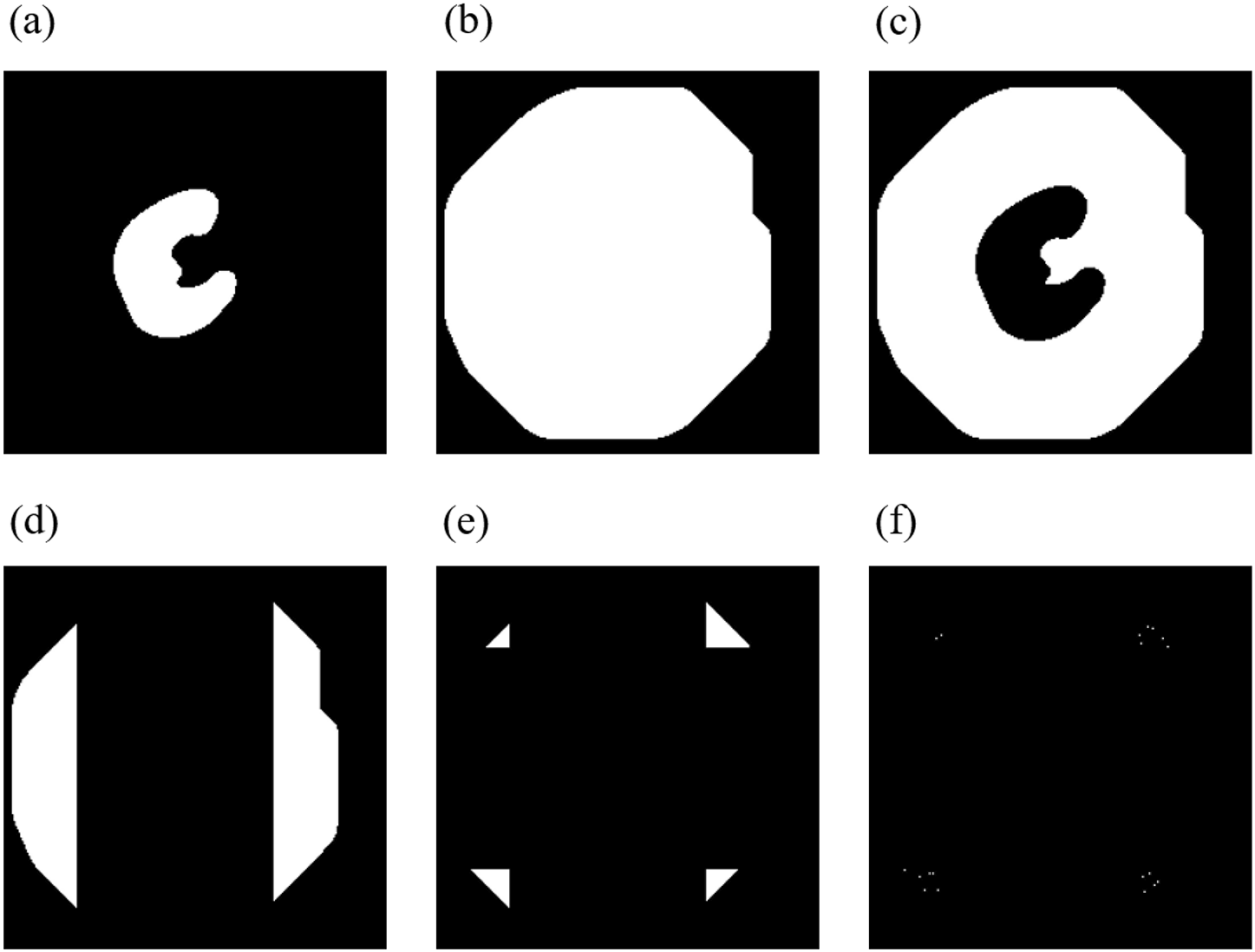
The process of generating points for the BC class. (**a**) Shows the kidney mask annotated by an expert. (**b**) Displays a mask enlarged with dilation transformation. (**c**) Shows the effect of overlapping and cropping the kidney mask from the image. (**d**) Shows the effect of strip removal on the Y axis. (**e**) Shows the effect of strip removal on the X axis. (**f**) Shows the final generated points.

**Figure 8:**
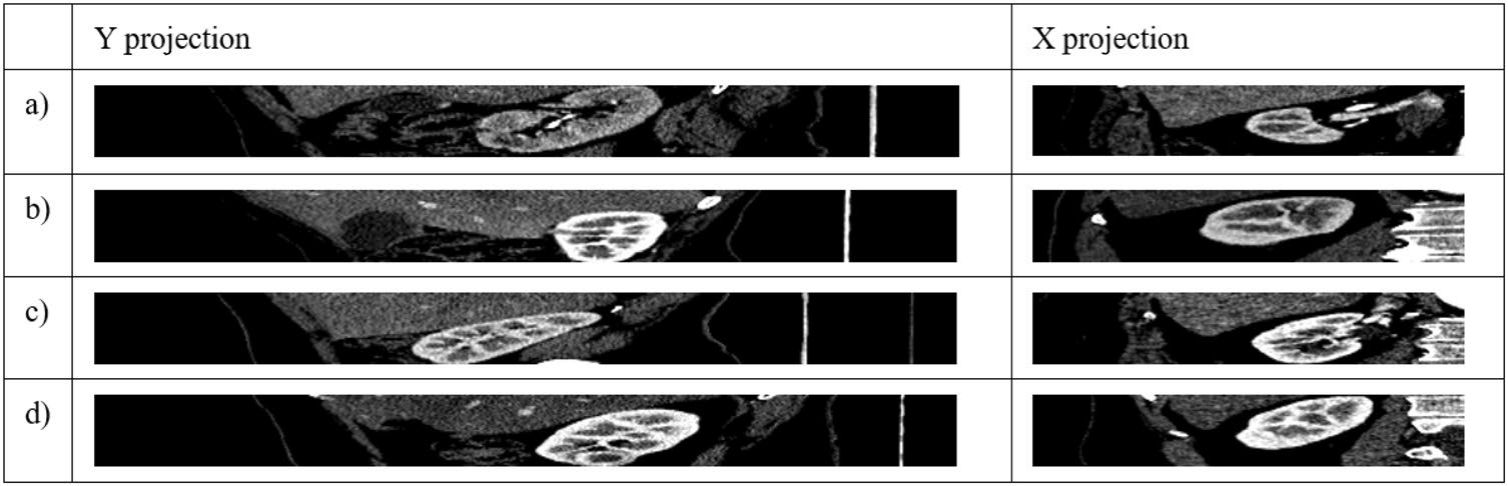
Example of four random images in X and Y projection for OC, resulting from point generation.

First, the binary mask previously prepared and labelled by an expert is dilated with a disk of radius r = 60 px and the indicated neighborhood of 4 (Fig. 7b). Then, the kidney mask is removed from the image (Fig. 7c). Also, the stripes on the X axis (Fig. 7d) and Y axis (Fig. 7e) are enlarged by 20 px in four directions (left, right, up, down). New points are generated from the created mask with a spacing of 50 px (Fig. 7f), just like OC. This way of point generation ensures that the points of class BC are sufficiently far away from the points of class OC and that no point of class BC intersects the kidney in the X and Y axes. In the next step, for each point generated, a cut is made in the X and Y axes for both classes (OC and BC). These cuts generate as many new images in the X projection and in the Y projection as there were generated points for the classes KC and BC. Figure 7 shows a representation of this process. An example of four random images in the X projection and in the Y projection for the class OC, resulting from point generation, is shown in Fig. 8.

**Figure 9:**
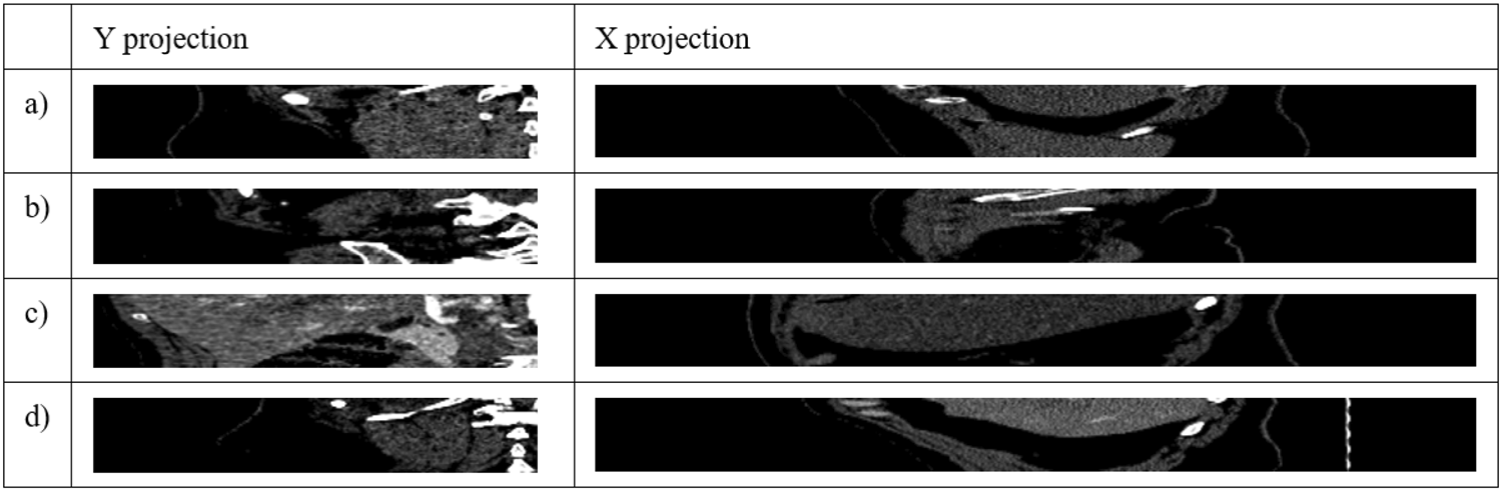
Example of four random images in X and Y projection for class BC generated by our solution.

Figure 9 shows four random images in X and Y projection for class BC generated by the method described earlier.

All generated images for both classes OC and BC are considered as learning data. The entire process is repeated analogously for the spleen.

#### Learning process based on CNN network

**Figure 10:**
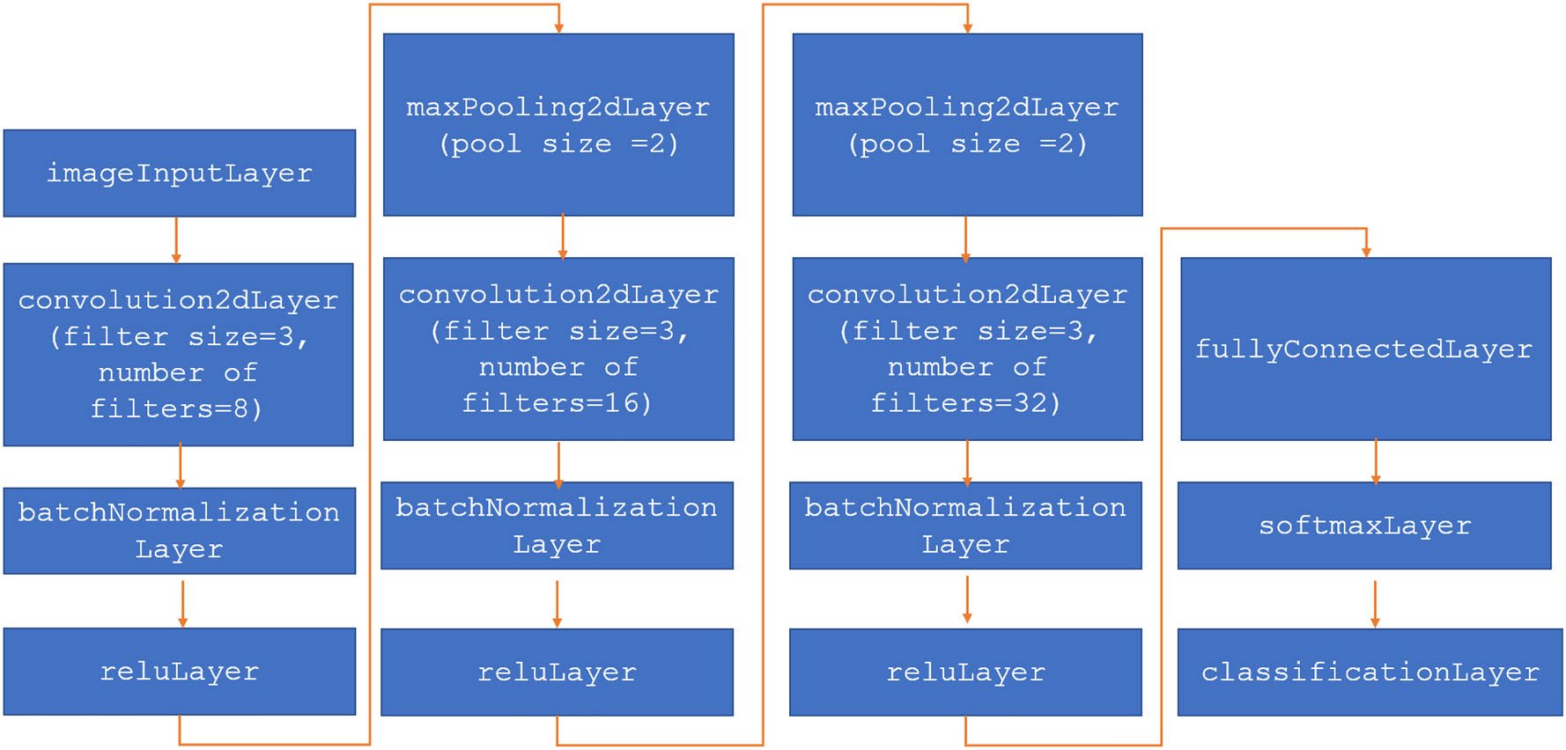
Complete architecture of the network used. An architecture consists of imageInputLayer, 2$\times$ convolution2dLayer, 3$\times$ batchNormaliza-tionLayer, 3$\times$ reluLayer, 2$\times$ maxPooling2dLayer, fullyConnected-Layer, softmaxLayer and classificationLayer.

In the first step of developing and training a convolutional neural network, the architecture must be defined. Convolutional neural networks were developed as tools for image analysis and recognition systems. They have eliminated the tedious and difficult phase of manual describing of image features. In this solution, the network itself is responsible for generating the features. Each layer of a CNN processes the image of the previous layer and looks for primitive features (e.g., groups of pixels with similar gray levels, edges, intersecting lines, etc.). Subsequent hidden layers produce certain generalizations of the features from the previous layer, organized in the form of images. In this work, we have chosen an architecture consisting of imageInputLayer, 2$\times$ convolution2dLayer, 3$\times$ batchNormalizationLayer, 3$\times$ reluLayer, 2$\times$ maxPooling2dLayer, fullyConnectedLayer, softmaxLayer and classificationLayer. The complete architecture of the network used is shown in Fig. 10.

In the final system, three networks are prepared: RoiNetX, RoiNetY and RoiNetZ, respectively for different X, Y and Z projections. The architecture of all networks is the same. However, they differ in the size of the input data. The size of RoiNetX is 43 $\times$ 512 $\times$ 1, RoiNetY is 43 $\times$ 256 $\times$ 1, and RoiNetZ is 512 $\times$ 512 $\times$ 1. These differences are due to the size of the images in the different projections. The size of the RoiNetZ network (512 $\times$ 512) is defined by the default CT image size. The size of RoiNetX (43 $\times$ 512) was chosen because the number of layers in the Z dimension is close to 43 for all learning data; otherwise, scaling is applied. RoiNetY (43 $\times$ 256) was prepared in the same way, but here the image was cropped to half (512/2), since only one kidney (left or right) is analyzed at a time. The training hyperparameters were established using random search and are as follow: solverName: Stochastic gradient descent with momentum (SGDM), InitialLearnRate: 0.01, MaxEpochs: 4, Shuffle: every-epoch.

#### Generation of test data

The test step (for an organ) requires the use of the three CNNs described in “Learning process based on CNN network”. Two networks: RoiNetX and RoiNetY, are used for clarity of algorithm representation, while the final system is based on three networks: RoiNetX, RoiNetY and RoiNetZ. A two-dimensional image is scanned for selected points, with a step of 10 px, starting from point (1, 1), which is the top left corner of the image. To optimize the algorithm, if RoiNetX does not detect a kidney at a given point $p(x_i,y)$, all points $p(x_1,y)...p(x_{max}Y)$ are excluded from the test. Similarly, if RoiNetY does not detect a kidney at a given point $p(x, y_i)$, all points $p(x, y_1)... p(x, y_{max})$ are also excluded from the test. By generating test points in this way, the search range for kidneys is significantly reduced. The following steps of the kidney detection process are shown in Fig. 10.

**Figure 11:**
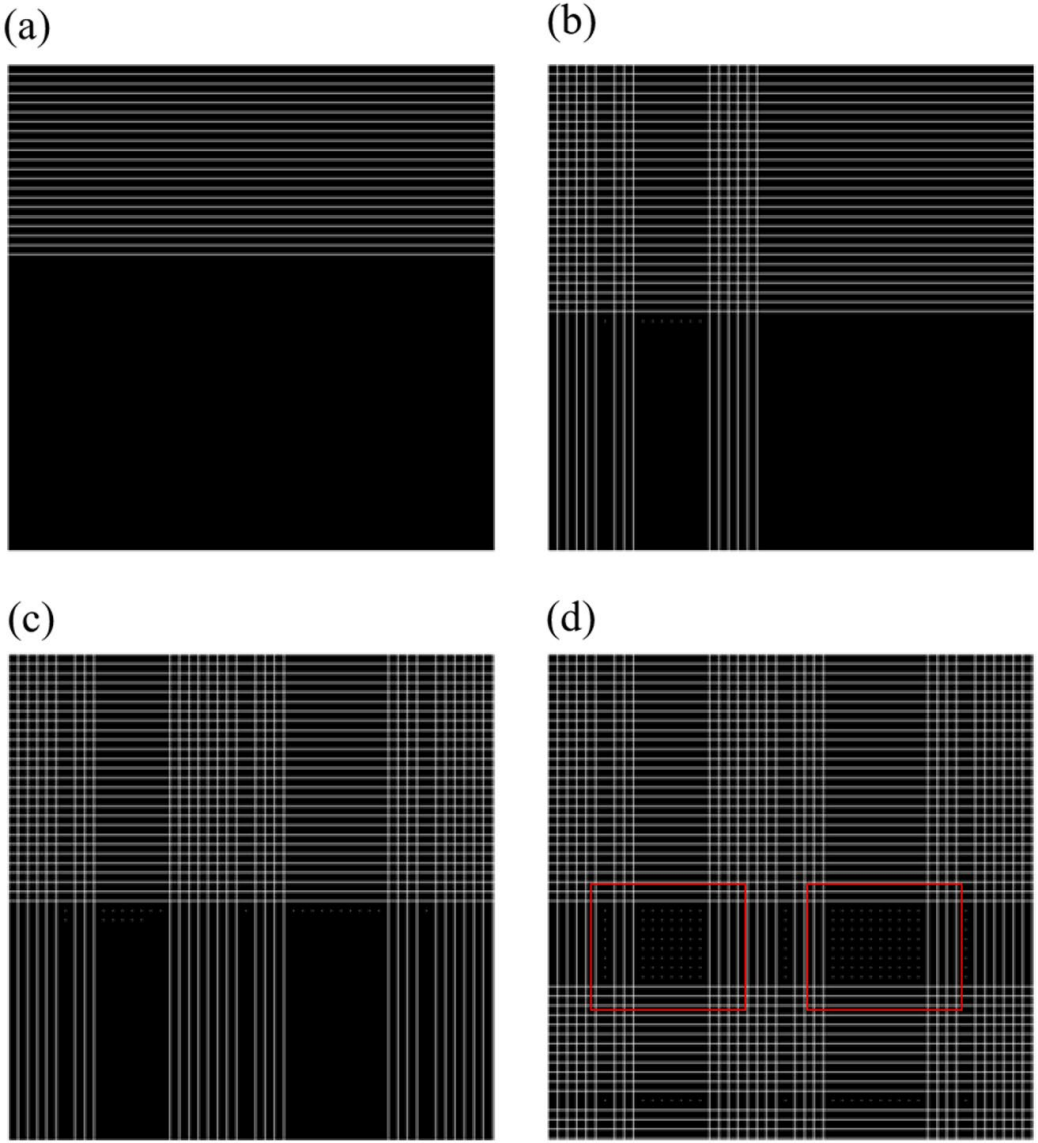
Visualization of the sequential steps of the kidney scan algorithm. White lines indicate areas already tested (or excluded).

Figure 11a–c show the successive steps of finding the region with the highest probability of kidney occurrence. White lines indicate pixels where kidneys were not found.

**Figure 12:**
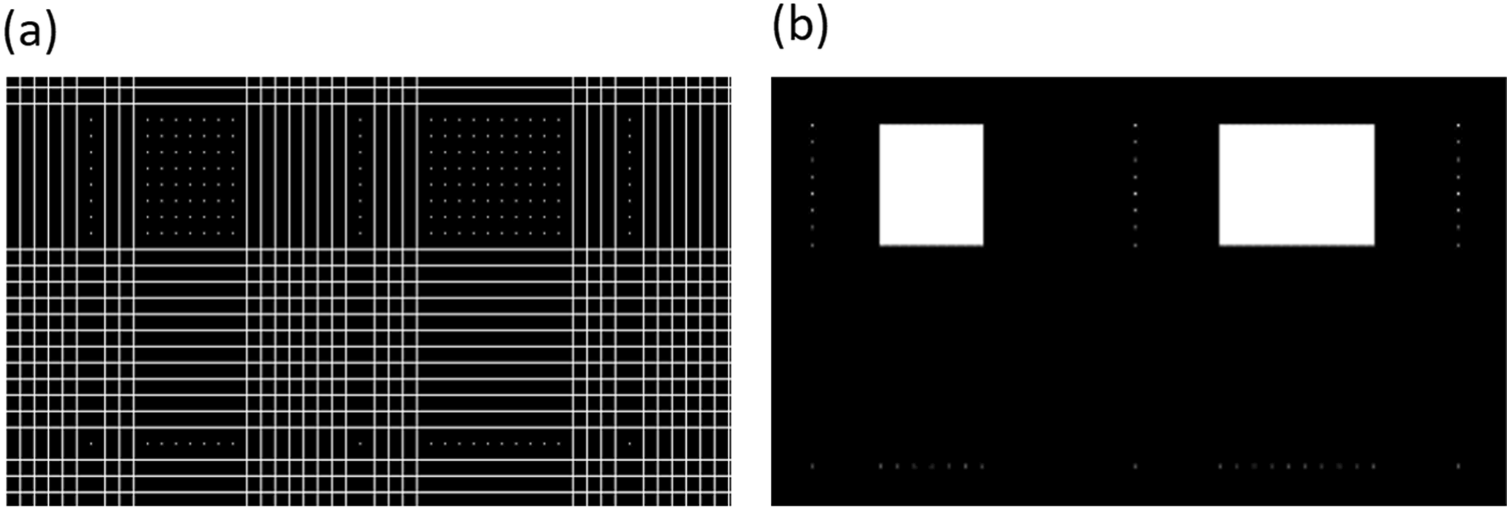
(**a**) Shows white lines defining areas excluded from classification, and white dots denoting areas classified as kidney (or spleen). (**b**) Shows the effect of merging equidistant points.

Figure 11d shows two rectangular (marked in red) areas that most likely contain the outline of the kidney. Figure 12a shows an en-largement of a portion of Fig. 11d. White lines indicate fragments that were excluded from classification, and white dots indicate pixels that were classified as kidney by both RoiNetX and RoiNetY together. The points arranged in a line in the lower part of Fig. 12a,b designate the misclassified points as kidneys. Figure 12b shows the effect of connecting equidistant points (with a value of 10 px) in the X and Y axes.

**Figure 13:**
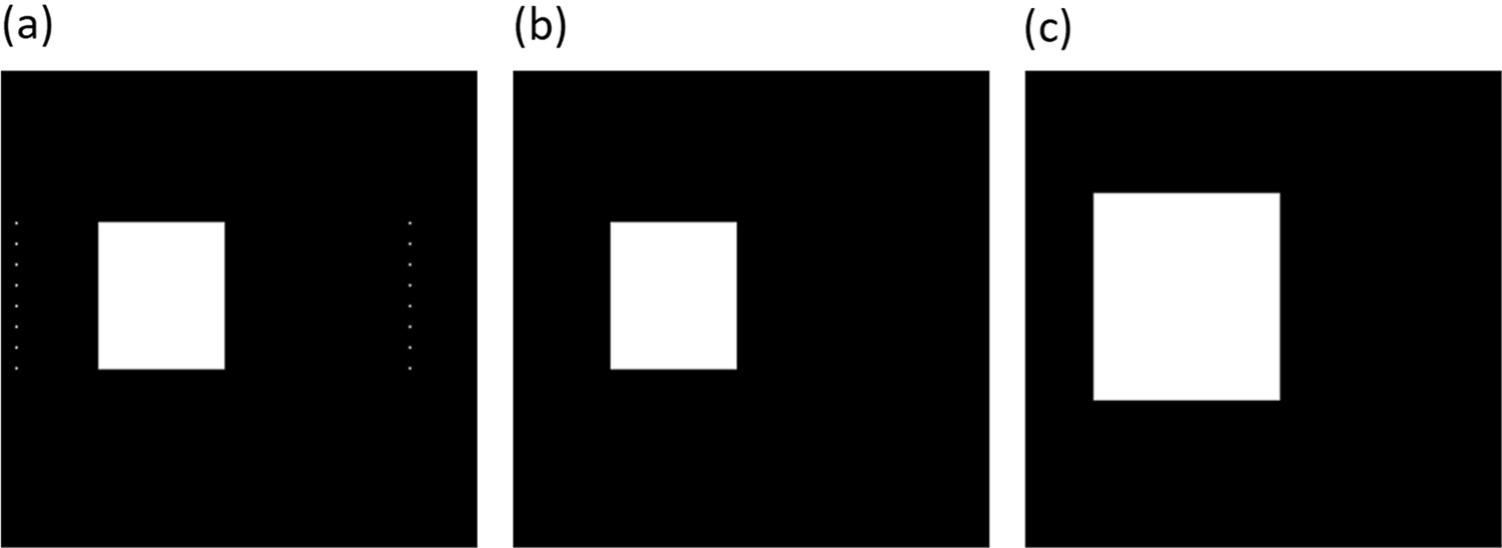
(**a**) Shows the circumcision on one organ. (**b**) Shows the morphological operation, leaving the largest areas. (**c**) Shows the result of the dilation operation using a structural element in the form of a square.

The next step is to perform a morphological operation in which only the largest area is preserved. A dilation operation is performed on a square structured object of 30 $\times$ 30 px to increase the ROI area. The results of the processes can be seen in Fig. 13.

**Figure 14:**
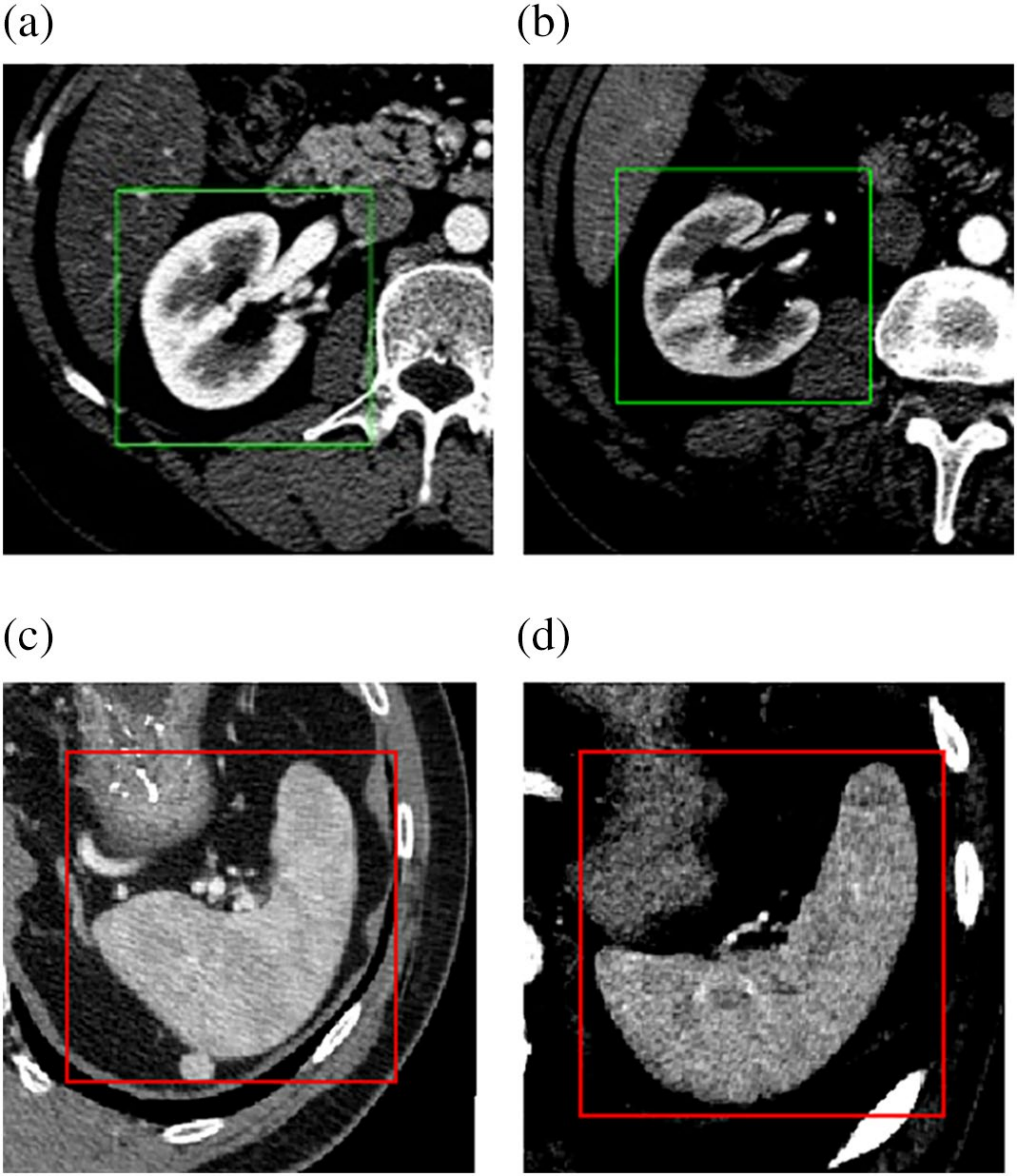
Example of four image slices with found ROI. Images (**a**) and (**b**) show recognition of the kidney, while images (**c**) and (**d**) show its counterpart on the spleen.

Figures 12b and 13a–c, clearly show the white rectangular areas that define the area of the kidney or spleen. The effect on the entire algorithm for ROI detection is shown in Fig. 14.

The final defined area containing the kidney or spleen is defined by a three-dimensional cube.

**Figure 15:**
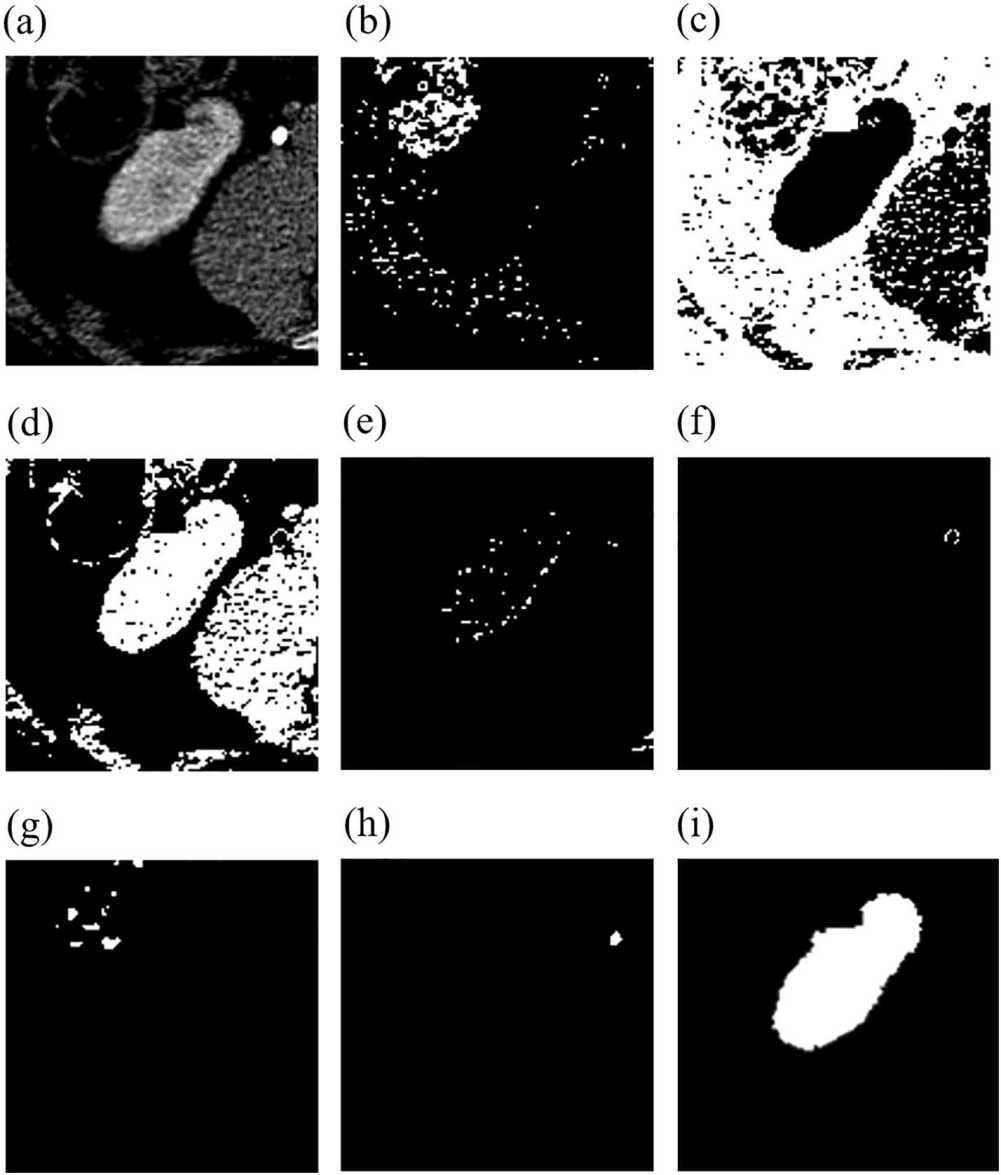
Example of region clustering algorithm. (**a**) Is the input image. (**b**–**h**) Are the automatically generated clusters. (**i**) Is the final computed kidney mask.

4.3 Having found, the ROI, we can proceed to the detection of the organ contours. For this purpose, an adaptive fuzzy K-means clustering algorithm has been implemented^31^. Clustering is performed only in the previously found ROI and divides the image into $n_c$ clusters, one of which is selected to represent the final kidney contour. All data is assigned to the nearest centers based on Euclidean distance. The new centers for the image with resolution R $\times$ S pixels are calculated using equation:

$$\begin{aligned} c_j=1/n_{c_j}\sum _{x\in c_j}\sum _{y\in c_j} p(x,y), \end{aligned}$$

where $x=1,2,...R$, $y=1,2,...S$, $j=1,2...n_c$, *p*(*x*, *y*) is the pixel under consideration and $c_j$ is the *j*-th center. The capabilities of the algorithm are shown in Fig. 15. The input data is subjected to clustering (Fig. 15a). In this case, we obtain seven clusters (Fig. 14b–h). Among all the clusters, the one whose center is closest to the center of the ROI is selected. Also, all areas outside the boundaries of the ROI are removed, and finally we perform a hole filling operation (Fig. 15i).

In Fig. 15, only one cluster (d) is selected from the seven clusters (b)–(h) and considered for further processing (filling holes, detecting a coherent region).

### Institutional review board statement

The study was approved by the Military Institute of Medicine in Warsaw, Poland (approval number 38/WIM/2017) Informed Consent Statement: All data used in the study is anonymized and there is no possibility of associating the study with a specific human. No additional human studies were required to complete the presented experiments. All tests were performed on archived CT images. We confirm that all participants have given informed consent for the study.

## Results

Numerical experiments were prepared to test the effectiveness of the proposed system. For each patient, experts from the Military Institute of Medicine in Warsaw manually contoured the kidneys and spleens. Using the previously selected learning data, the system generated a set of 3563 learning images. Then, more patients were selected to generate new test images (about 3000 for each case). Finally, the system fully automatically generated the final ROI regions for each organ (kidney and spleen). In each case, the ROI was successfully generated and completely contains the organ. The parameters used in the CNNs are listed in Table 1.

**Table 1:** The parameters used in the CNNs networks.

Parameter	Value
No of epoch	40
Learning algorithm	The stochastic gradient descent with momentum
InitialLearnRate	0.01
ValidationFrequency	30
Number of filters	32
Filter size	3
MiniBatchSize	64
Input size (RoiNetX)	43 $\times$ 512 $\times$ 1
Input size (RoiNetY)	43 $\times$ 256 $\times$ 1
Input size (RoiNetZ)	512 $\times$ 512 $\times$ 1

Clustering was performed only within the ROI. The result of the system is a set of binary masks representing the organ. The masks generated by the system were compared with the masks created manually by a human expert. Standard measures were used to evaluate the quality of organ contour segmentation in medical images^20,32,33^: true positive (TP), false positive (FP), false negative (FN), sensitivity (TPR) specificity (PPV) and F1-score (F1). The F1 score is defined in equation:

$$\begin{aligned} F1=(2\times {TP})/ (2{TP}+{FP}+{FN})\times 100\%, \end{aligned}$$

where TP denotes the number of pixels classified as kidney/spleen by both the system and the expert, FP denotes the number of pixels classified as kidney/spleen by the system but as background by the expert, and FN denotes the number of pixels classified as background by the system but as kidney/spleen by the expert. In addition, sensitivity and specificity were established as standard measures for medical system evaluation:

$$\begin{aligned} TPR=\frac{TP}{(TP+FP)}, \end{aligned}$$

and

$$\begin{aligned} PPV=\frac{TN}{(TN+FP)}. \end{aligned}$$

Table 2 contains cross-validation tests with average *TP*, *FP*, *FN*, *TPR*, *PPV* and *F*1 values for all cases divided into ten groups. Each group contains images obtained from 1 to 2 patients.

**Table 2:** Average values of *TP*, *FP*, *FN*, *TPR*, *PPV* and *F*1 to assess the accuracy of kidney contour detection.

Group	TP	FP	FN	TPR	PPV	F1
1	2827.99	271.27	439.90	86.16	92.21	88.88
2	2072.75	171.15	297.95	87.62	92.44	89.94
3	1825.00	97.53	371.63	82.96	95.26	88.65
4	2094.00	74.00	535.50	79.68	96.72	87.38
5	1407.00	192.80	147.80	90.02	88.24	89.02
6	2435.65	355.86	237.28	89.70	88.92	89.09
7	1709.73	149.23	291.59	84.49	93.44	88.60
8	1465.89	136.11	141.44	90.87	91.11	90.97
9	2361.64	239.41	270.91	89.88	91.39	90.53
10	2098.20	373.93	173.00	92.73	85.38	88.79
mean	2120.02	224.59	295.29	87.32	91.48	89.13

The average F1 score of the automatic system is 89.13% for kidney detection, which is a satisfactory result. Moreover, another test was performed to evaluate the effect of ROI detection on clustering ability. The results for kidney and spleen detection are shown in Table 3.

**Table 3:** Average F1 score for the cluster-based system before and after detection of the ROI. The results show the influence of the ROI on the final organ recognition.

	Kidney detection F1-score	Spleen detection F1-score
Proposed system based on ROI detecion	89.13	87.75
Clustering without using ROI	80.57	76.36

**Figure 16:**
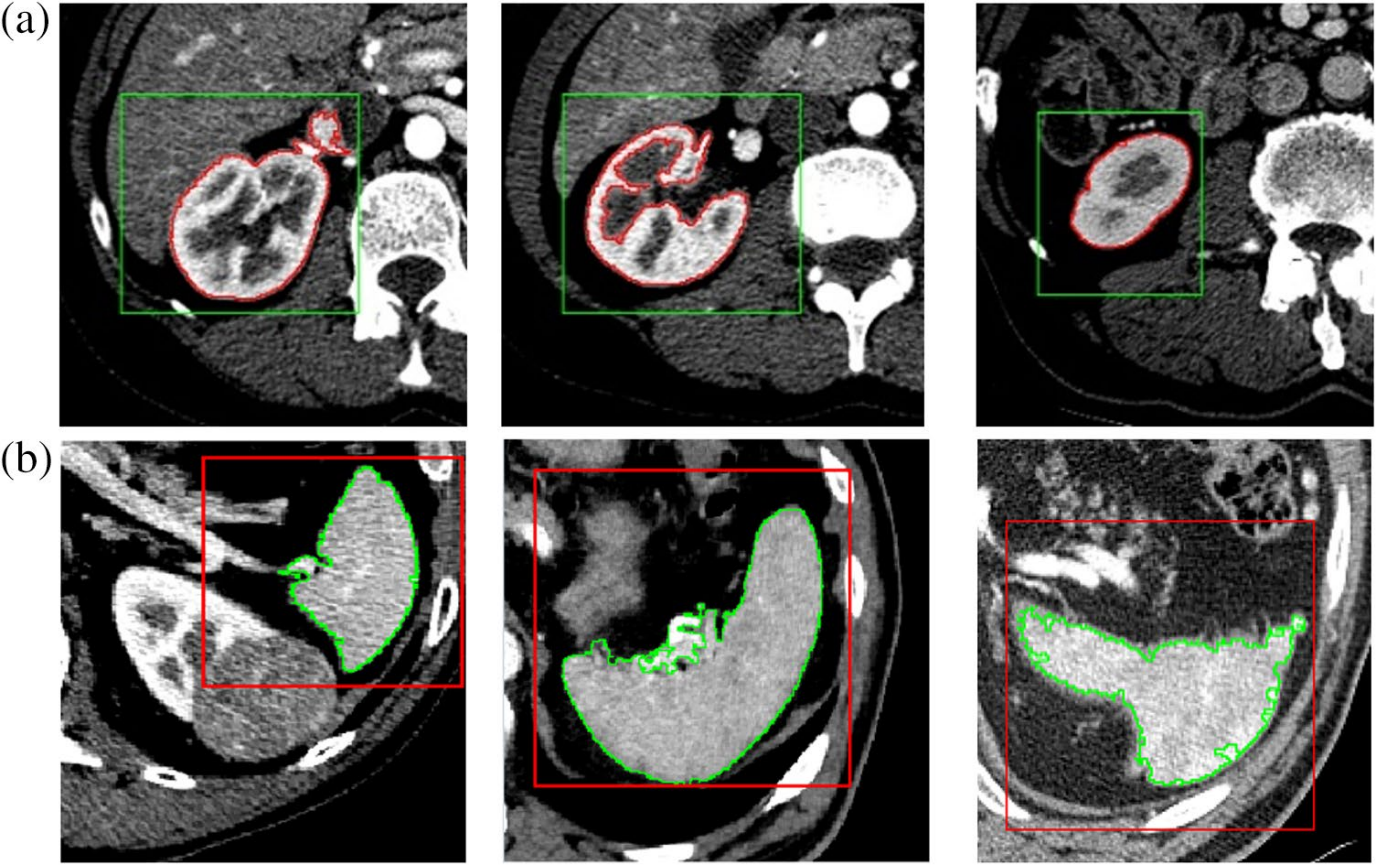
Examples of kidneys recognition. The rectangle indicates the detected ROI. The outline indicates the organ found: kidney (**a**) or spleen (**b**).

After implementing the initial ROI detection, a significant improvement in the accuracy of kidney and spleen contour detection is observed. The F1 score increased from 80.57 to 89.30% for kidney detection and from 76.36 to 87.75% for spleen detection. The average time to generate ROI for a case is 2.86 seconds in the tests running on Intel(R) Core(TM) i7-7820HQ CPU @ 2.90GHz, 2901 Mhz, 4 Core(s), 8 Logical Processor(s), Installed Physical Memory (RAM) 16.0 GB and Matlab environment. Figure 16 shows the visual results of kidney detection for selected kidneys.

Figure 16a shows the rectangular ROI in green. Within this ROI we used the organ detection procedure. The contour of the kidney was marked with red color. For the spleen, Fig. 16b, the detected ROI area was colored red and the spleen contour was colored green. The visual results confirm the high performance of the automatic system in detecting the kidney area.

The study has been implemented with segmentation evaluation metrics: Volumetric Overlap Error (VOE)—this method determines the percentage overlap between two images by using the Jaccard coefficient. Relative Volume Difference (RVD)—this method determines the difference between the reference image and the image after segmentation as a percentage Average Symmetric Surface Distance (ASSD)—this method is based on surface voxels that have at least one voxel outside the object in their neighbourhood. The Euclidean distance from the nearest voxel of the reference image is counted and averaged. Maximum Symmetric Surface Distance (MSSD)—also known as Haudorff distance, this method allows the difference between two sets of surface voxels to be determined using Euclidean distances. The maximum value from these distances gives the maximum symmetric surface distance. This method is particularly sensitive to boundaries and allows for a true maximum error^34^. These metrics allow for a more comprehensive evaluation of the performance of the segmentation algorithm, which allows for the proper selection of the segmentation algorithm and the adjustment of the algorithm parameters for best performance.

The proposed method was compared to popular alternative approaches: Graph-cut , U-Net 3D semantic segmentation and NNUnet framework.

Graph-cut is an automatic segmentation method that is often used for the detection of internal organs, such as kidneys, in computed tomography (CT) images. It involves using a graph optimisation algorithm to solve the problem of segmenting an image into two components: background and object.

In the graph-cut method, the image is treated as a graph consisting of vertices and edges. Each pixel is represented by a vertex of the graph, and edges between pixels represent the cost of the transition between them. Edges between neighbouring pixels have a low transition cost, while edges between far pixels have a high.

U-net is an automatic segmentation method that is often used for the detection of internal organs, such as kidneys, in computed tomography (CT) images. It is a convolutional neural network with a U-shaped architecture that consists of two parts: an encoder and a decoder. Another approach—U-net is designed to work with small datasets and its architecture allows it to learn representations of image features at different levels of abstraction, which is particularly important for kidney detection in CT images, as kidneys are relatively small objects relative to the whole image. U-net is also known for its good performance when segmenting irregularly shaped objects.

NNUNet is an extension of the 3D U-Net architecture, which is a deep learning model used for medical image segmentation. NNUNet also uses more advanced techniques for data augmentation, expanded architecture that includes more layers and a higher number of filters^35^.

Table 4 shows the mean VOE in %, RVD in %, ASSD in mm and MSSD in mm based on CT images for renal detection for 90 patients.

**Figure 17:**
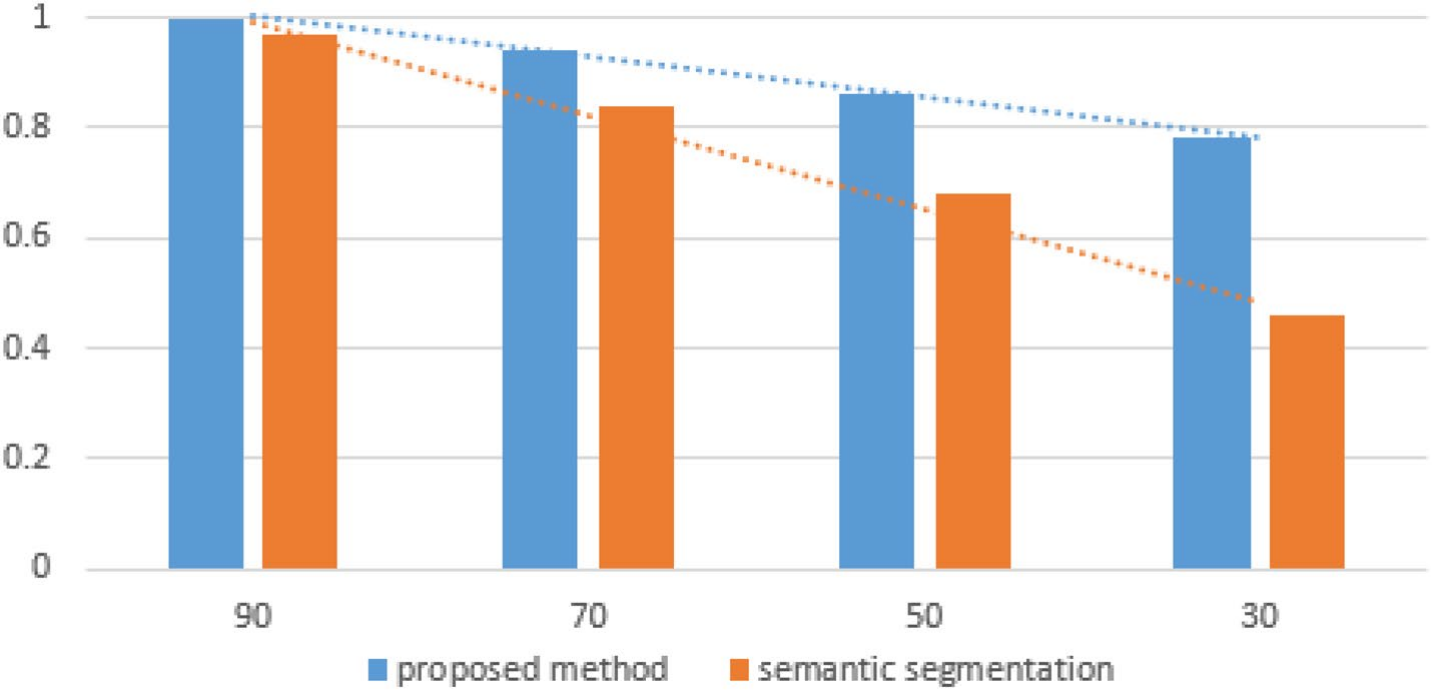
It is important to note that the CT object detection normalised accuracy for smaller datasets decreases significantly as the number of data decreases. For the proposed method, the trend line is less inclined.

It is important to note that the CT object detection accuracy for smaller datasets decreases significantly as the number of data decreases. Figure 17 shows a comparison of the trend line for the proposed method (based on classification) and semantic segmentation for different dataset sizes (data were split into learning and testing in prop 7/3).

Tests were also done on a benchmark dataset to check model training and testing times. The experiments were run on a low-cost computer equipped with an I5 processor, 4 cores, 6GB RAM. Two approaches were tested: semantic segmentation based on U-Net and the proposed method. The model training time for the proposed method is significantly lower: for the tested set of 60 patients, the learning time is 406 min, while for the proposed method it was 19 min. In the proposed method, additional pre-processing based on image processing methods had to be carried out, the total time of which was 4 min. In the end, a significant reduction in model learning time was achieved (406 min to 23 min) for the test dataset. The image testing times for semantic segmentation and the proposed method are quite similar. One 512 $\times$ 512 pixel image requires an average of 3.43 s for U-NET and 3.25 s for the proposed method.

**Table 4:** Mean values of segmentation evaluation error metrics: VOE, RVD, ASSD and MSSD based on CT images for renal detection.

	VOE (%)	RVD (%)	ASSD (mm)	MSSD (mm)
Proposed method	9.4	9.7	7.9	7.6
Graph-cut	15.6	17.6	19.42	17.9
U-Net 3D	9.7	9.8	7.8	7.8
nnUNet	9.6	10.0	7.7	7.7

**Figure 18:**
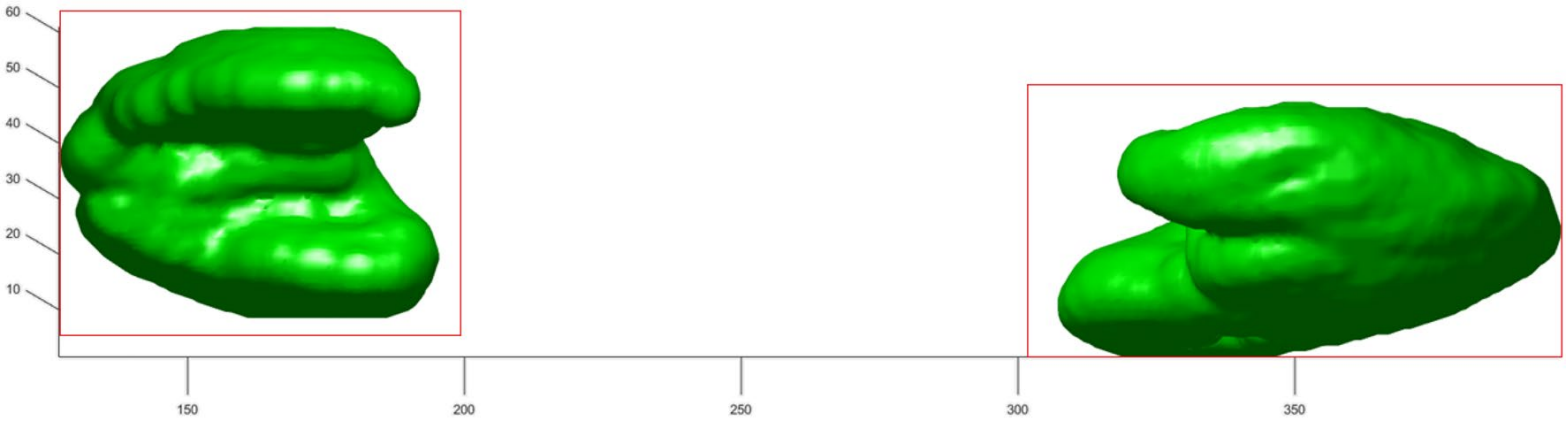
Precisely calculation of the ROI area allows for accurate segmentation of the organ.

The Fig. 18 depicts a 3D mesh of the segmented organ with the marked ROI area.

## Discussion

This paper presents a new method for detecting ROIs based on CNN classification applied to kidney and spleen recognition. Initial detection of ROIs is an important step in image processing and saves time by excluding unnecessary parts of the image from analysis. Currently, many works have used semantic segmentation (FCN) based on deep neural networks to find kidney regions^20,23,24,36^. This is now a standard procedure in medical image analysis and segmentation. Although semantic segmentation is a very convenient solution in terms of implementation, since it only requires the input masks of the search objects, it is characterized by high computational and memory complexity. To support small image sizes, e.g. 512 $\times$ 512 pixels, additional computational units, e.g. GPUs, are often required. Although the FCN model saves memory and computation time by optimizing the processing of overlapping regions for sliding windows, similar to CNN there is still a need to find new solutions that are easily accessible on standard CPU-based units. Recently proposed semantic segmentation models such as YOLO can now be run on a CPU. However, for the deployment of some solutions such as a U-Net for medical imaging^37^, the use of a GPU is recommended. Not every medical center can afford expensive hardware. Another major advantage of the proposed technique is that it can be applied to whole body scans of the abdomen to find scans that contain a kidney or spleen segment. Many kidney segmentation works omit or simplify this process, assuming that the scans containing the organ to be analyzed are known in advance. The proposed method can also be used in the detection of other renal or splenic abnormalities, especially renal lesions such as tumors. We are currently working on the possibility of using the technique for the detection of other organs such as the liver or pancreas. Another challenge in automatic organ segmentation is the ability to detect overlapping and touching organ boundaries. The contrast differences between organs are small and typical morphological methods fail. The proposed technique opens new possibilities in multiple organ segmentation. The main advantage of our technique is the ability to automatically detect multiple organs with a common approach with high efficiency. Our solution does not require pre-filtering of CT scans and the algorithm can accept the entire set of abdominal slices as input. Some drawbacks of our approach are the higher complexity of the system implementation (the need to generate learning and test images based on previously computed points), the need to use three networks instead of one network for each organ, and the post-processing in case of a complete organ contour detection. However, these disadvantages do not mask the important advantages of our solution: lower hardware requirements (no GPU needed, lower CPU and RAM requirements than typical U-Net based algorythms), lower learning time (up to 17$\times$ faster), higher efficiency for limited data sets, modularity—allowing easier parallelization of calculations. With standard neural network-based solutions, we don’t have so many options for parallelizing computations—and options are often limited to multithreading. Our solution can be more easily parallelized—each of our networks can be run independently on different computing units and the results easily merged. A table comparing advantages of our solution in comparison to U-Net based methods in four categories: Hardware requirements, Training and testing time, Size of data sets, and Segmentation accuracy is presented in Table 5. The system presented in this paper is successfully used in kidney and spleen contour detection and performs the task with 88–89% F1 score, which is a satisfactory result. Based on the numerical results, the influence of ROI segmentation on image clustering ability could also be investigated. The developed system will improve the quality medical specimen assessment by providing accurate quantitative assessment (pre-operative and pre-surgical), which is crucial for deciding the treatment method of a patient. The proposed system will speed up the process of accurate diagnosis by a physician.

**Table 5:** A table comparing advantages of our solution in comparison to U-Net based methods in four categories: hardware requirements, training and testing time, size of data sets, and segmentation accuracy.

	Proposed method	U-Net based metods
Hardware requirements	Low hardware requirements (no GPU requirements, tested on 4 cores)	High requirements (GPU required, 4G of VRAM, At least 6 CPU cores)
Learning time	Less learning time (17$\times$ time faster)	Greater learning time (grows significantly with larger data sets)
Testing time	Comparable	Comparable
The number of datasets	Good efficiency with limited data set size	Significant decrease in efficiency with small datasets. The need for large learning datasets
Segmentation accuracy	Comparable	Comparable
Modularity	Easy paralization on diffrenet computing units	Paralization based on multithreading

## Conclusions

The analysis of medical images is a time-consuming task, and it is necessary to constantly develop new computer methods for processing medical images based on modern solutions such as neural networks. Computer-aided medical diagnosis makes it possible to obtain an increasingly accurate diagnosis in a shorter time. Further development of organ recognition systems is necessary as further exploration of larger data sets using a fusion of methods. Despite the challenging task, the proposed approach allows to obtain the contours of the kidneys and spleen at a satisfactory level and can be the basis for the implementation of a practical system functioning in medical centers specialized in diseases of the abdomen.

## Data Availability

The data that support the findings of this study are available from Military Institute of Medicine in Warsaw but restrictions apply to the availability of these data, which were used under license for the current study, and so are not publicly available.
